# A large forage gap in forage availability in traditional pastoral regions in China

**DOI:** 10.1016/j.fmre.2023.01.003

**Published:** 2023-01-12

**Authors:** Tong Yang, Jinwei Dong, Lin Huang, Yuzhe Li, Huimin Yan, Jun Zhai, Jie Wang, Zhenong Jin, Geli Zhang

**Affiliations:** a College of Land Science and Technology, China Agricultural University, Beijing 100193, China; b Key Laboratory of Land Surface Pattern and Simulation, Institute of Geographic Sciences and Natural Resources Research, Chinese Academy of Sciences, Beijing 100101, China; c Satellite Environment Center, Ministry of Ecology and Environment of the People's Republic of China, Beijing 100094, China; d College of Grassland Science and Technology, China Agricultural University, Beijing 100193, China; e Department of Bioproducts and Biosystems Engineering, University of Minnesota, St. Paul 55108, MN, United States of America

**Keywords:** Forage supply-demand, Natural grassland, Crop straw, Green fodder, China, Provincial

## Abstract

Forage supply has been stressed due to the rapid increase in China's livestock consumption. However, the long-term dynamics of the relationships between forage demand and multi-sourced supply are not understood. Here, we examine the annual forage demand, or practical carrying capacity (PCC), and supply, or theoretical carrying capacity (TCC) from 2000 to 2019 in China. We construct a forage supply-demand index (FSDI) to represent the forage supply pressure using MODIS-derived net primary productivity products and provincial statistical datasets, and we consider two scenarios. First, natural grasslands are the sole source of forage. Second, natural grassland forage supply is supplemented with straw crops. We find an increase in PCC in northwestern China's major pastoral and agropastoral provincial regions, including Inner Mongolia, Gansu, Ningxia and Qinghai, at rate of 0.24-3.59 million sheep units (SU) a year. As the primary source of forage, the theoretical carrying capacity of natural grasslands (TCC_grass_) expanded at a rate of 3 million SU/yr nationally. Crop straws fed 126.58 million SU nationally in 2019, which accounted for 11.3% of the total practical carrying capacity and alleviated the forage supply pressure by reducing FSDI by 26.56%. During 2000–2019, the theoretical carrying capacity of straw crops (TCC_crop_) increased rapidly from 76.5 million SU to 126.6 million SU, which accounted for 10%-15% of the total forage supply at the national scale. We also discovered large carrying capacity gaps (TCC_gap_) in the northwestern pastoral provincial regions of Inner Mongolia, Xinjiang, Gansu, and some agricultural provinces such as Shandong and Henan, when we considered forage supply from both natural grasslands and straw crops. Our findings showed a large forage gap in the traditional pastoral regions, and we also discussed green fodder as a potential solution for balancing the supply of and demand for forage, which may shed light on crop and forage planning.

## Graphical abstract

**Figure:**
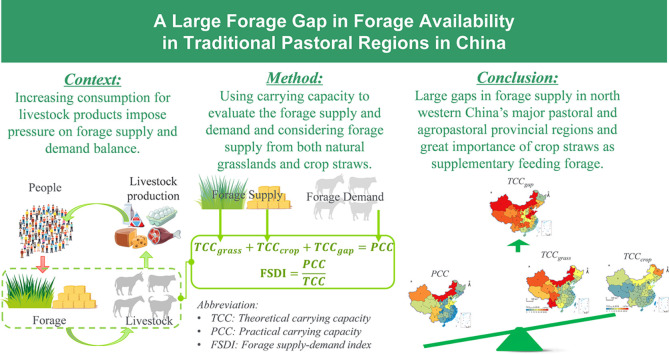

## 1 Introduction

For the world's most populous countries, such as China, food security is critical. People's dietary choices are changing due to the remarkable improvement in national living standards [1,2], as evidenced by decreased consumption of grain and increased demand for livestock products (e.g., meat, eggs, and milk) [3], [4], [5]. According to a previous study, per capita demand for animal-sourced calories in China is expected to rise 23% between 2020 and 2050 [6], which will place additional strain on livestock production. A consistent forage supply is critical for grass-based livestock husbandry and food security [7], and currently, natural grasslands are the primary source of forage in China [8,9]. For instance, natural grasslands in the mid-western provinces accounted for 46.75% of the total land area and 92.03% of total national grasslands, which play an important role in the provision of ecosystem services, including forage supply, biodiversity, and soil carbon sequestration [5]. Due to the increasing grazing pressure, natural grassland degradation is widespread in China [8], which threatens grassland sustainability and national food security [10]. The Chinese government has implemented the Grazing Exclusion Projects to restore grassland ecosystems, but these projects may aggravate the conflict between forage supply and demand [9]. Climate change may also exacerbate the forage-livestock imbalance [11].

Forage is mainly comprised of the edible parts of plant leaves and stems, and it includes not only plants eaten directly by livestock, such as pasture grass, but also straw crops, immature cereal crops, or similar plants that are harvested as fodder and fed to animals as hay or silage [8,11]. Diversifying forage sources, such as supplemental feeding and ground-feeding with sown pastures, straw crops, forage crops, dual-purpose crops, and shrub-based pastures [12], has been advocated in some studies to reduce grazing pressure on natural grasslands and increase the availability, accessibility, and affordability of forage [13].

Current research has generally focused on assessing regional forage-livestock balance using only natural grasslands [14], [15], [16], and few studies have investigated multiple sources of forage (straw crops and green fodder) and its regional differences. Also, previous studies have been conducted in traditional pastoral areas, especially in ecologically vulnerable areas, such as Tibetan Plateau, and arid and semi-arid areas, such as Inner Mongolia and Xinjiang [17], [18], [19]. However, national-scale investigations are urgently needed, especially in non-traditional pastoral areas and farming-pastoral areas, where land competition between humans and livestock is more severe [20]. Therefore, a comprehensive understanding of forage supply and its different sources at the national scale would be of significnce for identifying the hotspots with the conflict between forage supply and demand.

Remote sensing based net primary productivity (NPP) datasets have been widely used for estimating crop yield and vegetation productivity due to their multi-year fine spatiotemporal resolution observations [21]. For example, Xin et al. (2019) analyzed the grassland ecosystem carrying state and its changes based on the NPP supply and consumption in a Kazakhstan grassland system [16]. Huang et al. (2020) estimated the forage yield using the NPP of grassland derived from the Carnegie-Ames-Stanford approach (CASA) and grassland biomass data from field research, and then evaluated the grassland pressure based on practical and theoretical carrying capacity [8].

The carrying capacity of grassland is derived from the concept of the population carrying capacity [22,23], and refers to the number of livestock that a certain area of grassland can carry in a period without causing damage to natural resources and the environment [24]. Here, we aimed to investigate the forage supply-demand relationship in China as characterized by carrying capacity at the provincial scale during 2000–2019 using remote sensed multi-year NPP datasets and statistics. Our objectives were multiple: (1) to understand forage demand and its changes as measured by practical carrying capacity (PCC); (2) to estimate the carrying capacity of only natural grasslands (TCC_grass_) using MODIS-derived NPP data and to evaluate the forage supply-demand balance state using the forage supply-demand index considering only natural grasslands (FSDI_grass_); (3) to estimate the carrying capacity of straw crops (TCC_crop_) using statistical data from the provincial official statistical yearbook and evaluate the forage supply-demand balance state by considering both natural grasslands and straw crops (FSDI_crop__+grass_); and (4) to understand the forage supply structure, including natural grasses, straw crops , and green fodder among provinces and introduce a pertinent forage supply development strategy. We then discussed the potential for achieving local forage supply-demand balance by expanding forage resources, such as green fodder.

## 2 Materials and methods

We assessed forage supply and demand in China and on national and provincial scale from 2000 to 2019 using carrying capacity as a consistent indicator to eliminate the ambiguity caused by the units used by our various data sources. Here, we focused on forage for herbivorous livestock like cattle, horses, donkeys, mules, camels, goats, and sheep. Herbivorous livestock is mainly fed on natural grasslands in traditional pastoral provincial regions, such as Xinjiang, Inner Mongolia, Qinghai, and Tibet. These areas are dominated by grassland- based livestock production systems (GL) [25]. Recently, various mixed farming-livestock husbandry methods have been adopted in traditional pastoral regions, such as supplementary feeding with crop straw, harvested grasses, green fodder, and cultivated forage crops [8,26]. In the central and eastern provinces, house feeding is very common, which are classified as mixed rainfed farming livestock production systems (MR) and mixed irrigated farming livestock production systems (MI) according to the livestock production system classification scheme of Food and Agriculture Organization (FAO) (Fig. S5) [25,27].

Here, we considered both grassland and farming- based systems, because livestock production systems are not completely monocultures, which means different proportions of GL, MR and/or MI co-exist in one province. On the demand side, we used practical carrying capacity (PCC) to characterize the total demand for forage, which is the actual number of livestock carried by a certain area within a certain period and reflects the present livestock carrying demand (Section 2.1). On the supply side, we used the theoretical carrying capacity (TCC) and considered two sources of forage, which were natural grasslands (TCC_grass_) and straw crops (TCC_crop_). TCC is the maximum number of livestock that a certain area can carry in a certain period to meet the needs of normal growth, reproduction, and production of livestock under the premise of moderate grazing and sustainable grassland production [28,29].

TCC_grass_ characterized the forage supply capacity of local natural resources controlled mainly by climate change (Section 2.2) [14]. We quantified the forage supply capacity of straw crops (TCC_crop_) as artificial supplementary feed (Section 2.3). Also, we constructed an index of forage supply and demand (FSDI) using the quotient of practical and theoretical carrying capacities to demonstrate carrying pressure in each province. By comparing the differences in FSDI without and with straw crops as supplementing forage supply, we quantified the effect of straw crops as a potential way to alleviate grazing pressure of natural grasslands (Section 2.4). Finally, we estimated the carrying capacity gaps (TCC_gap_) in each province (Section 2.5). Here, our workflow followed the state-of-the-art national calculation standard in the *Calculation of rangeland carrying capacity* [28,30], which was issued by the Chinese Ministry of Agriculture in 2015. The carrying capacity unit is a standard sheep unit (SU). All the different types of livestock were converted into one adult sheep weighing 45 kg and consuming 1.8 kg of standard hay per day according to the corresponding ratio [28].

### 2.1 Total demand for forage characterized by the practical carrying capacity (PCC)

PCC was calculated from the mid-year and year-end livestock numbers using Eqs. 1-3. We considered the major large livestock included in the statistical scope of the yearbook of each region, which included cattle, horses, donkeys, mules, camels, goats, and sheep [31,32]. All the livestock were converted to a standard unit of SU [28].

(1) $$\text{PCCyear}=Stock_{y,Mid}\times \frac{T_{warm}}{365}+Stock_{y,End}\times \frac{T_{cold}}{365}$$

(2) $$\mathrm{PC}C_{\mathrm{warm}}=\mathrm{Stoc}k_{y,\mathrm{Mid}}=\mathrm{Stoc}k_{y,\mathrm{End}}+\mathrm{Slau}\mathrm{ghte}\mathrm{rs}=\mathrm{Stoc}k_{\mathrm{End}}+\mathrm{Slaughter}\;\mathrm{Rate}\times \mathrm{Stoc}k_{y-1,\;\mathrm{End}}$$

(3) $$\text{PCCcold}=Stock_{y,End}$$

where $Stock_{y,Mid}$ is the mid-year population of livestock for a given year y, $Stock_{y,End}$ is the population of livestock at the end of the year y, $T_{warm}$ is grazing days in the warm season, and $T_{cold}$ is grazing days in the cold season. We assumed grazing activity occurs throughout the year, so here $T_{warm}$ is warm season days and $T_{cold}$ is cold season days, i.e., $365-T_{warm}$. $T_{warm}$ depends on the length of growing season for natural grasslands [33]. The detailed description for the methodology for determining the $T_{warm}$ was shown in Text S1.

### 2.2 Supply of natural grassland characterized by theoretical carry capacity (TCC_grass_)

We estimated the TCC_grass_ by using a remote-sensed vegetation net primary production (NPP) dataset and the grassland resource type dataset. NPP is the quantity of net organic dry matter accumulated by plants under natural conditions after photosynthesis and respiration [34,35]. The natural grassland dataset was a completion of field surveys and grass production measurements in more than 2000 counties in China and was then verified and supplemented by aerial and satellite imagery [36]. The following steps outline our calculations.

First, we estimated the total yield of natural grasslands (Y, kg/m^2^) using remote-sensed vegetation NPP dataset from MODIS (MOD17A3HGF V6) using Eq. 4. Considering the availability, regeneration and utilization of natural grasslands, the available forage yield was estimated and then converted into standard hay yield (F, kg/m^2^) based on 19 subtypes of natural grasslands across China (Eq. 5) [36]. Hay harvested at the peak of the growing season with 14% moisture content in temperate grassland or mountain steppe and meadow is considered standard hay [37]. Forage yields of 19 subtypes of grasslands were unified as yield of standard hay [29]. The detailed parameters including grassland subtype, root/shoot ratio, regeneration rate, utilization rate , and the standard hay conversion coefficient of grassland are shown in Table S1. Then, we converted the standard hay yield into theoretical carrying capacity per area of natural grasslands (TCC_1_) using Eq. 6. To demonstrate the total carrying capacity of natural grasslands (TCC_grass_), we aggregated TCC_1_ based on natural grasslands area in each provincial region using Eq. 7.

(4) $$Y=\;\frac{NPP}{t\left(1+r\right)\times \left(1-WaterContent\right)}$$

where Y is the total yield of natural grassland per area (kg/m^2^), t is the coefficient of biomass conversion to productivity approximated as a constant 0.45 [8], r is the root/shoot ratio of plants, and *WaterContent* is set to a constant 14%.

(5) $$F=Y\times \left(1+a\right)\times UR_{ng}\;\times K$$

where F is available standard hay yield per area (kg/m^2^), a is the regeneration rate of natural grasslands, $UR_{ng}$ is the utilization rate of natural grasslands, and K is the standard hay conversion coefficient of grasslands.

(6) $$TCC_{1}=\frac{F}{Q_{d}\times T}$$

where $TCC_{1}$ is the theoretical carrying capacity of natural grasslands per area (SU/m^2^), $Q_{d}$ is standard hay intake for one sheep unit per day (1.8 kg/SU per day), and T is grazing days of one year and assumed as 365.

(7) $$TCC_{grass}=\sum TCC_{1}\times Pixel\;Area\;$$

where $TCC_{grass}$ is the theoretical carrying capacity of natural grasslands.

### 2.3 Supply of crop straw characterized by theoretical carry capacity (TCC_crop_)

Crop straw is widely used as domestic animal feed [38], and straw-based roughage is an important component of ruminant livestock nutrition [26]. Here, we calculated the amount of crop straw resources using the ratio of above-ground stalk yield to economic yield of crops (i.e., the straw to grain ratio [39],Eq. 8), and then estimated the theoretical livestock carrying capacity (TCC_crop_) using the straw feeding ratios of different crops [26] (Eq. 9). The detailed datasets are shown in 2.7 and the parameters are shown as Tables S2-S3.

(8) $$W_{s}=W_{P}\times S_{G}$$

where $W_{s}$ is straw production of crops measured as fresh weight (kg), $W_{P}$ is the economic production of crops (kg), and $S_{G}$ is the straw-to-grain ratio.

(9) $$\mathrm{TC}C_{\mathrm{crop}}=\frac{W_{s}\times f}{Q_{f}\times T}$$

where f is straw feeding ratio, and we referred to the existing literature for each province (Table S3). $Q_{f}$ is fresh forage intake for one sheep unit (4 kg/SU per day), and T is the number of grazing days of 365.

### 2.4 Forage supply and demand balance

We constructed FSDI to demonstrate the balance state of forage supply- demand using practical and theoretical carrying capacities as follows:

(10) $$FSDI=\frac{PCC}{TCC}$$

where FSDI is the forage supply and demand balance index. Here we evaluated the FSDI under two scenarios, natural grass supply only (FSDI_grass_) and natural grass and crop straw together (FSDI _crop+grass_). The denominators of the FSDI formulas for the two scenarios were TCC_grass_ and TCC_grass_ + TCC_crop_, respectively. The FSDI grading criteria was shown in Table 1 [8,16].

**Table 1:** **The grading standard for FSDI**.

Forage supply-demand state	Surplus	Basic balance	Mild imbalance	Imbalance	Moderate imbalance	Severe imbalance
FSDI	<0.5	0.5–1.5	1.5–3	3–5	5–10	≥10

### 2.5 Forage gap considering both natural grasslands and straw crops

We estimated the forage supply gap based on actual demand (PCC) and known supplies (TCC_grass_ and TCC_crop_) following Eq. 11. The production gap of forage was calculated by multiplying the theoretical grazing days (T) of 365 and dry forage intake for one sheep unit 1.8 kg/SU per day (*Q_d_*) using Eq. 12.

(11) $$TCC_{gap}=\text{PCC}-TCC_{grass}-TCC_{crop}$$

(12) $$\text{Forage}\;\text{gap}=TCC_{gap}\times T\times Q_{d}$$

### 2.6 Analysis of the degree of variation and trend

We used the coefficient of variation and linear regression trend to reflect the changes in supply and demand of forage over the past 20 years. The coefficient of variation (CV) is the ratio of the standard deviation to the mean, which indicates the degree of dispersion of a collection of data and can eliminate the impact of data dimension. Here we calculated the CV of PCC, TCC, FSDI, and other indicators from 2000 to 2019 to reflect their variability over the past 20 years. The magnitude of the CV value represents the degree of variation in the indicators during 2000–2019 (see Table 2) [40,41]. The trend analysis from 2000 to 2019 was characterized as the slope of the linear least square regression of the interannual variation of indicators, which included PCC, TCC_grass_, TCC_crop_, FSDI_grass_, FSDI_crop+grass_, TCC_gap_, and green fodder area (Eq. 13). Also, the p-value was estimated to suggest the significance of linear regression [42].

(13) $$Slope=\;\frac{n\times \sum {}_{i=1}^{n}\left(i\times I_{i,k}\right)-\sum {}_{i=1}^{n}i\sum {}_{i=1}^{n}I_{i,k}}{n\times \sum {}_{i=1}^{n}i^{2}-{\left(\sum {}_{i=1}^{n}i\right)}^{2}}$$

where i is the order of year from 1 to n, and n is the number of years; $I_{i,k}$ is the indicators’ value.

**Table 2:** **The grading standard for CV**.

Variation degree	Weak variation	Moderate variation	Strong variation
CV	0–0.15	0.15–0.35	≥0.35

### 2.7 Materials

#### 2.7.1 MODIS NPP product

We obtained the annual NPP from MOD17A3HGF V6 during 2000–2019, at 500 m pixel resolution (https://lpdaac.usgs.gov/products/mod17a3hgfv006/). The annual NPP is derived from all 8-day Net Photosynthesis (PSN) products from MOD17A2H from the given year [43]. The PSN value is the difference of the gross primary productivity and the maintenance respiration. The dataset has cleaned the poor-quality inputs from 8-day leaf area index and fraction of photosynthetically active radiation (LAI/FPAR) based on the quality control (QC) label for every pixel [43]. The MOD17 NPP products have been validated with eight biome types including tallgrass prairie and desert grassland round the world and showed no overall bias [44]. MOD17A3 NPP product has been applied to investigate yields due to its fine resolution and spatial-temporal continuity [43,45,46]. For example, Reeves et al. (2006) compared the NPP product and filed measurements of peak biomass from grasslands in Oklahoma and confirmed that the MODIS NPP products are suitable for regional grassland studies [47]. Later, Jan de Leeuw et al. (2019) used the MODIS NPP product to assess the carrying capacity of grassland in Azerbaijan and confirmed that this product was reliable for estimating carrying capacity [45]. Wei et al. (2022) used the MOD17A3HGF dataset to analyze the spatiotemporal variation in NPP in Shaanxi Province, China, which implied that this dataset was suitable to assess regional vegetation productivity of China [46].

#### 2.7.2 Natural grassland map

The Grassland Resource Map of China at a scale of 1:1000,000 (GRMC) defined 19 natural grassland subtypes and their spatial distribution [36]. This dataset reflected the subcategories of natural grasslands by integrating vegetation and the environment of pastoral, semi- agricultural, and semi- pastoral regions. The classification scheme adopted the vegetation-habitat multi-factor classification method, following the principles of occurrence, economic utilization, and stability of grassland types. Therefore, this dataset was suitable for delineating the different natural environments of China and has been used for many multi-year investigations [8,48]. The dataset was downloaded from Resource and Environment Science and Data Center.

#### 2.7.3 Statistical datasets and other datasets

For our study, we obtained the numbers of herbivorous livestock and crop production in each provincial region during 2000–2019 from official statistical yearbooks published by the national and provincial governments, including provincial statistical yearbooks (1999–2020), the Statistical Data of Agriculture in People's Republic of China for 60 Years [32], and the China Agriculture Statistical Report (1999–2017) [31]. The straw-to-grain ratios and straw feeding ratios were obtained from relative research with reliable results (Tables S2-S3).

## 3 Results

### 3.1 Spatiotemporal pattern of changes in livestock

**Fig. 1:**
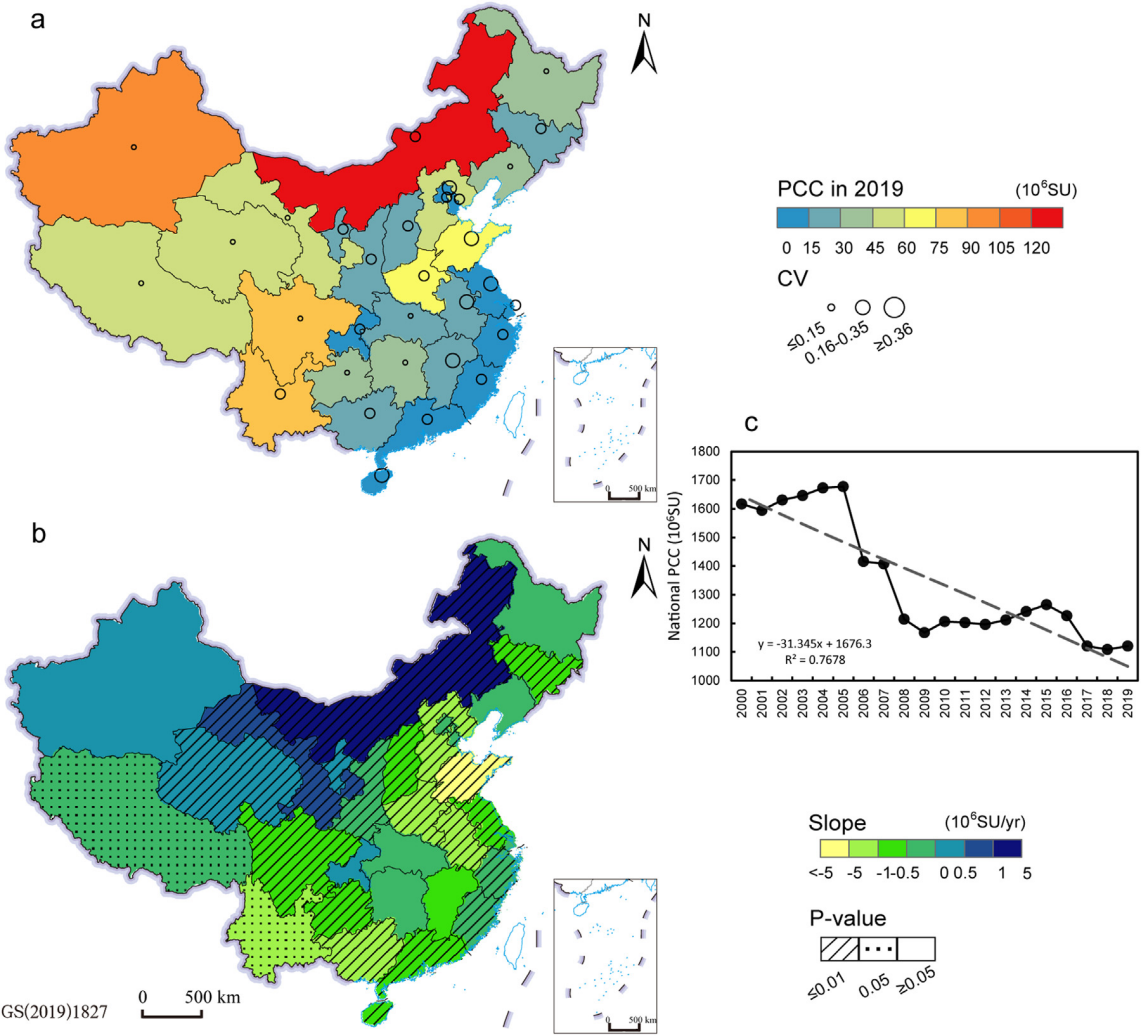
**Practical carrying capacity (PCC) in 2019 and its changes.** (a) is the provincial PCC in 2019 and coefficient of variation (CV) during 2000–2019. (b) is the dynamic trends of provincial PCC during 2000–2019 and its corresponding p-value. (c) is the dynamic trend of PCC in the national scale during 2000–2019.

Western pastoral provincial regions showed greater demand for forage than other regions during 2000–2019. The PCCs in western arid and semi-arid provincial regions were higher, especially in Inner Mongolia where the PCC in 2019 was greater than 120 million SU (Fig. 1a). And these regions also had significant positive trends in PCC, especially Inner Mongolia (3.59 million SU/yr), Gansu (0.92 million SU/yr), Ningxia (0.36 million SU/yr), and Qinghai (0.24 million SU/yr) (Fig. 1b). While the PCC in southern China was smaller than 45 million SU and decreased significantly during 2000–2019 (Fig. 1b). We noted that the status quo of PCC was relatively high in central China, such as in Henan and Shandong, but showed a significant negative trend during 2000–2019 (Fig. 1a, b). Nationally, PCC had a negative trend over the 20 years (−31 million SU/yr), and the largest demand for forage occurred in 2005 (1.68 billion SU) and the smallest demand occurred in 2018 (1.11 billion SU) (Fig. 1c).

### 3.2 Forage demand-supply balance considering natural grasslands only

**Fig. 2:**
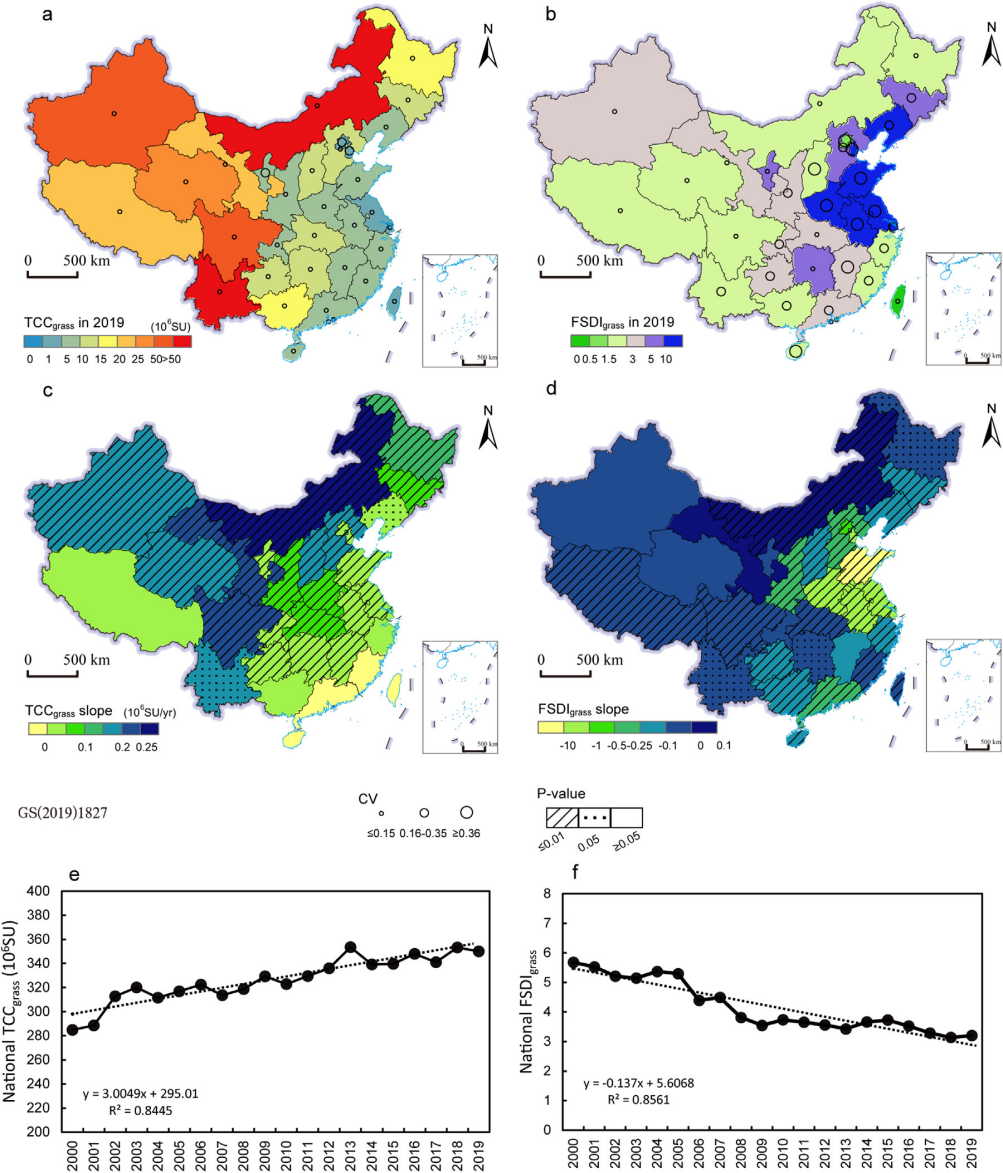
**Theoretical carrying capacity of natural grasslands and the forage supply-demand index (FSDI) considering only natural grasslands.** (a, c) are the provincial TCC_grass_ in 2019, coefficient of variation (CV) and dynamic trends and its corresponding p-value during 2000–2019. (b, d) are the provincial FSDI considering only natural grasslands in 2019, coefficient of variation (CV) and dynamic trends and its corresponding p-value during 2000–2019. (e, f) are the national TCC_grass_ and FSDI_grass_ and their changes during 2000–2019.

We found a positive trend in theoretical carrying capacity of natural grassland per area in most of China, with the largest increases being concentrated in the ecological restoration area of the Green for Grain Project (GGP), especially in central and eastern Inner Mongolia, Gansu, and northern Shaanxi (Fig. S1). However, we found a negative trend in TCC per area in southern China (Fig. S1). The provincial total TCCs supplied by natural grasslands (TCC_grass_) were relatively high in mid-western traditional pastoral provinces due to the vast area of grassland. Specifically, Inner Mongolia, Sichuan, Yunnan, and Xinjiang produced more than 51.6% of China's total natural grassland forages (Fig. S2) and fed more than 181.66 million SU livestock (Fig. 2a). The supply of natural grasslands in these provincial regions grew smoothly during 2000–2019 and was characterized by weak variability and a significant positive trend in TCC_grass_ (Fig. 2a, c). In the central and eastern provinces of China, the TCC_grass_ were lower, and most of the provinces had a slight upward trend, while individual provinces (Guangdong and Fujian Provinces) had a downward trend in TCC_grass_ (Fig. 2a, c). Meanwhile, nationally, the TCC_grass_ of the country increased at a rate of 3.00 million SU/yr (Fig. 2e).

However, we found the local forage demand cannot be satisfied by only natural grasslands, but the deficiency was alleviated with the growth of TCC_grass_. The local forage demand considerably outstripped local supply at the provincial scale in 2019, as evidenced by the FSDIs exceeding 1.5 when natural grasslands were the only forage supply (Fig. 2b). The most serious imbalance occurred in some eastern provinces, such as Henan, Shandong, Anhui, Jiangsu, and Liaoning, followed by some agropastoral provinces (FSDI_grass_ between 5 and 10), such as Ningxia, Jilin, and Hebei Provinces. In the Tibetan Plateau provinces, the FSDI_grass_ values were minimal (<3, Fig. 2b). We found that the FSDI_grass_ had a downward trend in most provinces, except for Inner Mongolia and Gansu (Fig. 2d). Given that the TCC_grass_ in Inner Mongolia and Gansu significantly increased over the last 20 years (Fig. 2c), the increased FSDI_grass_ indicated a deficiency in the local forage supply of natural grasslands. Nationally, FSDI_grass_ had a fluctuating downward trend during 2000–2019 (Fig. 2f).

### 3.3 Forage demand-supply balance considering both natural grasslands and straw crops

To quantify the effects of straw crops as a supplementary feeding source, we calculated the total crop straw production of main crops, which included paddy rice, wheat, corn, sorghum, millet, and soybean (Table S2), then estimated theoretical carrying capacity of straw crops (TCC_crop_) and evaluated the forage supply-demand relationships under supplies from natural grasslands and straw crops (FSDI_crop+grass_).

**Fig. 3:**
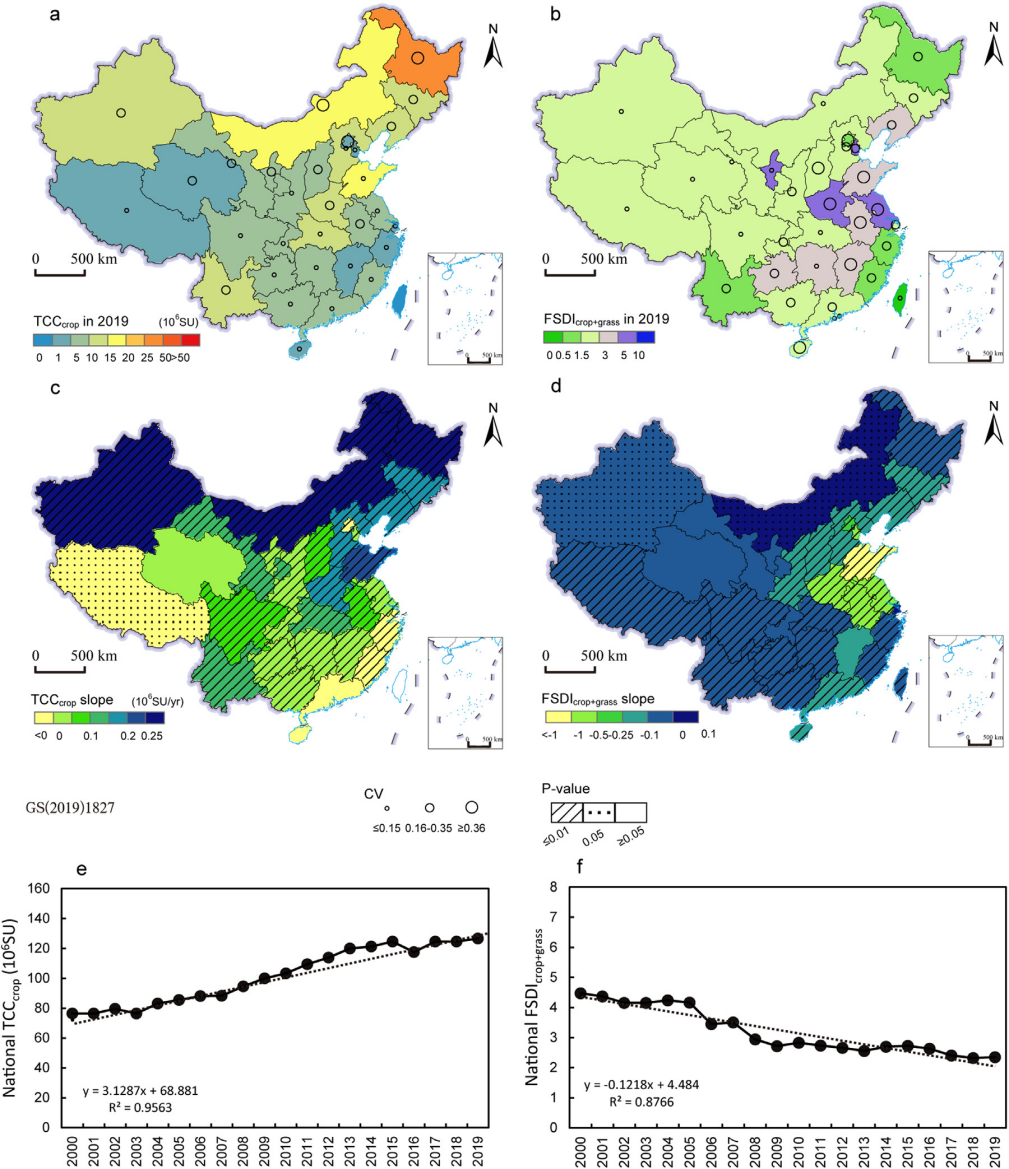
**Theoretical carrying capacity of crop straw (TCC**_**crop**_**) and the forage supply-demand index considering natural grasslands and crop straw (FSDI**_**crop+grass**_**).** (a, c) are the provincial TCC_crop_ in 2019, coefficient of variation (CV) and dynamic trends and its corresponding p-value during 2000–2019. (b, d) showed the provincial FSDI_crop+grass_ in 2019, coefficient of variation (CV) and dynamic trends and its corresponding p-value during 2000–2019. (e, f) are the national TCC_crop_ and FSDI_grass+crop_ and their changes during 2000–2019.

We observed that the forage supplied by straw crops was not always consistent with the production of crop straw, because the supply depends on several factors, such as the production of crop grain, the straw feeding ratio, and straw proportion of crop plants. By comparing the production of crop straw in 2019 and the straw feeding ratio (f), we found that although crop straw production was high, the proportion of straw for feed was relatively low in China (less than 50%, Fig. S3b, Text S2). Nonetheless, the carrying capacity of straw crops still played an important role in the mixed farming livestock production systems, which was reflected by the increasing TCC_crop_ and decreasing FSDI_crop+grass_. Fig. 3 showed the theoretical carrying capacity of straw crops (TCC_crop_) and the FSDI considered natural grasslands and straw crops (FSDI_crop+grass_). Crop straws could feed 20.34 million SU nationally in 2019, or one third of the TCC_grass_ (Figs. 2e and 3e).

We found that Heilongjiang Province produced the most crop straw (854.2 billion kg, Fig. S3a), and TCC_crop_ was the largest with over 20.36 million SU in 2019, followed by Shandong (10.96 million SU) and Inner Mongolia (10.51 million SU) (Fig. 3a). Moreover, we found that the TCC_crop_ has increased during 2000–2019 except Tibet (Fig. 3c), especially in Heilongjiang Province, increasing at a rate of 0.78 million SU/yr. The TCCs supplied by straw crops in Inner Mongolia and Xinjiang Province also increased rapidly at the rate of 0.41 and 0.26 million SU/yr, respectively (Fig. 3c).

The FSDI more intuitively reflected the indispensable role of crop straw, especially in non-traditional pastoral regions. Compared to the FSDI for only natural grasslands (Fig. 2b), crop straw alleviated forage supply and demand pressure for most provinces, especially for Shandong and Anhui Provinces where the FSDI changed from being a severe imbalance (FSDI_grass_> 10) to a general imbalance (FSDI_crop+grass_ is bewteen 3 and 5) (Fig. 3b). In Xinjiang, Gansu and Shaanxi Provinces, the imbalance of forage supply- demand changed to mild imbalance (from 3.63 to 2.91, 3.28 to 2.59 and 4.24 to 2.62, respectively) after considering both straw crops and natural grasslands.

Overall, the forage supply-demand state was generally positive, with a significant decrease in FSDI_crop+grass_ over the last 20 years but was less optimistic in traditional pastoral regions especially Inner Mongolia. The FSDI_crop+grass_ in Inner Mongolia increased at an estimated rate of 0.02 per year (Fig. 3d). Nationally, the carrying capacity of straw crops showed a continuous upward trend (Fig. 3e), which may have contributed to the decrease of FSDI_crop+grass_ (Fig. 3f). Our findings suggested that making full use of crop by-products may alleviate the local forage supply pressure without increasing the grazing intensity of natural grasslands.

### 3.4 Differences in forage structure between provinces

Although crop straw alleviated the forage-livestock contradiction, we found large spatial heterogeneity in forage supply structure among provincial regions, and large gaps remained in mid-western forage supply system when both natural grasslands and straw crops were considered.

**Fig. 4:**
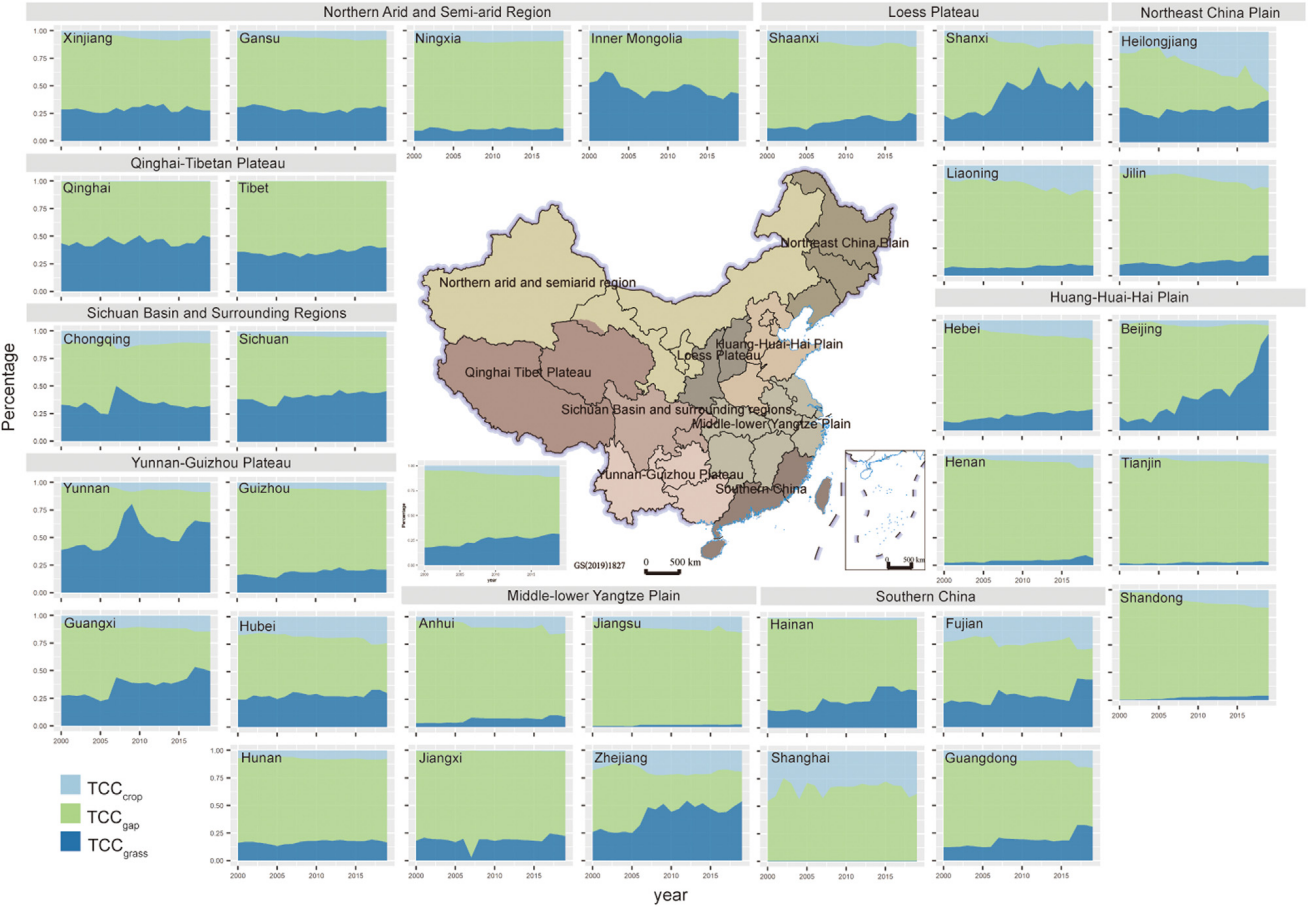
**Percentage of TCC supplied by natural grassland (TCC**_**grass**_**), straw crops (TCC**_**crop**_**), and the carrying capacity gap (TCC**_**gap**_**) estimated by practical carrying capacity (PCC) minus TCC**_**grass**_**and TCC**_**crop**_**for each province and the whole country during 2000–2019.** The center map is the nine agricultural subregions of China from the Resource and Environment Science and Data Center (https://www.resdc.cn/data.aspx?DATAID=275). Here the agricultural subregions were only for display.

Fig. 4 showed the relative proportions of TCC_grass_, TCC_crop,_ and TCC_gap_ over the 20 years. TCC_gap_ was calculated by subtracting the TCC_grass_ and TCC_crop_ from the PCC in each provincial region. Nationally, the proportion of TCC_grass_ accounted for about 20–30%, and TCC_crop_ accounted for about 10–15% of the total forage supply, and both have been increasing over the 20 years. Generally, the proportion of TCC_grass_ dominated the forage supply system in the Northern arid and semi-arid region, Qinghai-Tibetan Plateau, Sichuan Basin and surrounding regions, Yunnan-Guizhou Plateau, and Loess Plateau (Fig. 4). In these provinces, TCC_grass_ contributed about 50% of the forage supply on average with the minimum contribution of around 12.5% (Ningxia) and the maximum contribution of 75% (Yunnan). Among the provincial regions, we found notable upward trends in the share of TCC_grass_ in some provinces, such as in Guangxi, Shanxi, Shaanxi, Beijing, Hebei, Zhejiang, and Guangdong, where the increases ranged from around 12.5% to 75%. These were regions where key national ecological projects were implemented, such as Grain for Green, the Beijing-Tianjin Sandstorm-Control Program, the Grassland Ecological Restoration and Reconstruction Projects. We also found a significant increase proportion of TCC_crop_ in northeastern China of Heilongjiang, Liaoning, and Jilin Provinces, and in the Huang-Huai-Hai Plain of Hebei, Henan, and Shandong Provinces, where the increases ranged from around 12.5% to 25%.

**Fig. 5:**
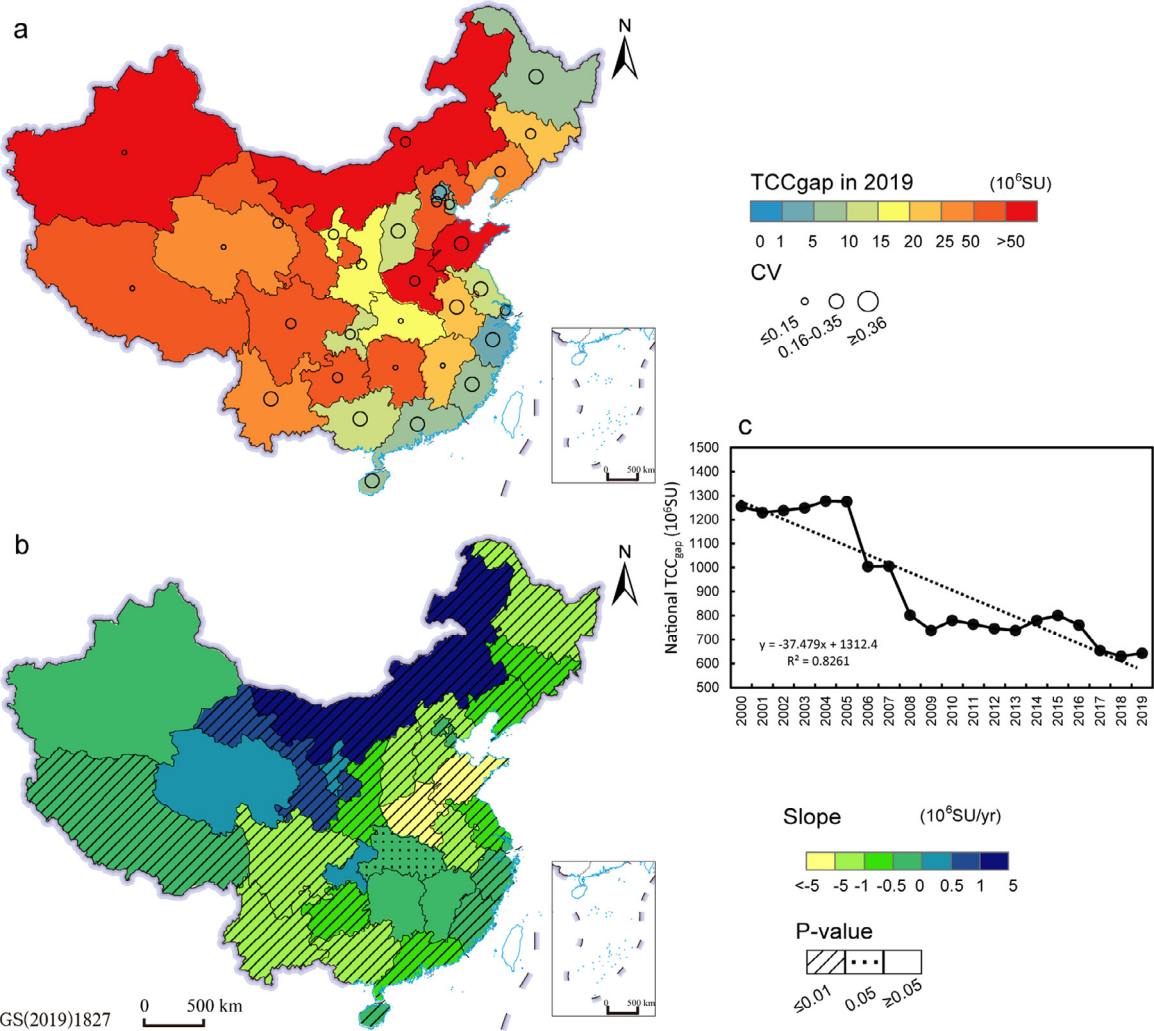
**Theoretical carrying capacity gaps in 2019 and changes.** (a) is the provincial TCC_gap_ in 2019 and coefficient of variation (CV) during 2000–2019. (b) is the dynamic trends of provincial TCC_gap_ during 2000–2019 and its corresponding p-value. (c) is the national total TCC_gap_ during 2000–2019 and its dynamic trend.

However, the huge gaps in theoretical carrying capacity cannot be ignored. Only Heilongjiang, Yunnan, Zhejiang, and Fujian Provinces balanced forage supply and demand in 2019. Fig. 5 shows the forage gaps and patterns characterized by TCC_gap_. Specifically, the carrying capacity gaps in the mid-western pastoral provincial regions were large in 2019, especially in Inner Mongolia and Xinjiang, where TCC_gap_ was up to 70 and 26.5 million SU in 2019 (Fig. 5a), which accounted for 49.37% and 65.7% of the provincial PCC (Fig. 4), respectively. Large gaps were also found in Shandong and Henan where the TCC_gap_ was 54.04 and 52.43 million SU in 2019 (Fig. 5a), which accounted for 80.0% and 84.5% of the provincial PCC (Fig. 4). Conversely, in Shanxi, Heilongjiang and southeastern provinces, the TCC_gap_ were relatively lower in 2019, ranging from 0.89 million SU in Zhejiang to 9.76 million SU in Guangxi (Fig. 5a), but the shares of TCCgap in PCC varied from 6.8% in Heilongjiang to 83.0% in Jiangsu (Fig. 4). These high shares implied not only higher external dependencies on forage supply but also higher ecological pressure on natural grasslands in these provinces.

During 2000–2019, we noted that the variability of TCC_gap_ was higher in eastern China than in the western provinces over the 20 years (Fig. 5a) but had different trends. The TCC_gap_ in Inner Mongolia, Gansu, and Ningxia had significant upward trends of 2.21, 0.56 and 0.29 million SU/yr, respectively, while Shandong and Henan had significant downward trends of 17.45 and 5.19 million SU/yr (Fig. 5b). In southeastern provinces, the downward trends were relatively weaker (Fig. 5b). Nationally, the carrying capacity gap fluctuated downward with a trend of -37.48 million SU/yr (Fig. 5c).

## 4 Discussion

Here, we used the carrying capacity as the uniform metric to demonstrate forage supply and demand, and we constructed the FSDI to reflect the forage supply- demand pressure and its changes over the past 20 years.

### 4.1 The forage supply and demand

We found that there were large gaps in forage supply in pastoral regions even though many ecological restoration projects were implemented. Since 2000, a series of ecological protection and restoration measures, including the use of livestock enclosures, reducing livestock numbers, returning farmland or rangeland to grassland, restoring severely degraded grasslands, and controlling grassland rodents, have been implemented in traditional grazing areas such as Inner Mongolia, Gansu and Qinghai-Tibetan Plateau [8]. The TCC_grass_ per area in these regions generally increased over the past 20 years, which was consistent with the conclusions in the latest publicly available national grassland monitoring reports [49].

A study on the Three-River Headwater (TRH) region showed that the grazing pressure was alleviated due to increased grassland yield and decreased livestock numbers during 2003–2012 [15]. We also found that the TCC_grass_ increased at an average rate of 0.46 million SU/yr in the northwestern provincial regions, which included Qinghai, Inner Mongolia, and Gansu during 2000–2019 (Fig. 2). However, the increase in natural grassland carrying capacity was not sufficient to meet the actual demand for livestock, and large gaps in theoretical carrying capacity were found in these regions (Fig. 5). Our findings agreed with other studies that also found that natural grasslands remained overstocked in ecologically fragile areas despite nearly a decade of ecological restoration projects, and that the grazing pressure had significant spatial heterogeneity [15].

Also, the forage supply of natural grasslands was easily affected by climatic factors and natural disturbances, such as precipitation [50], fire, or wild herbivores and woody plant encroachment [51]. In eastern and south-eastern China, we found relatively steady and slightly downward trends in TCC_grass_ (Fig. 2), which was probably due to the small area of natural grasslands and rapid urbanization [52]. Therefore, the forage supply of natural grasslands was extremely unstable and varied regionally, which implied that a great challenge remains to achieve a regional balance in forage supply and demand when relying only on natural grasslands.

We also found that crop straw has high potential as an additional forage source by comparing the FSDI_grass_ and FSDI_crop+grass_. Previous research has shown that concentrate supplementation improved the health condition of livestock, increased profitability, and lowered economic risk, and year-to-year variability [53], [54], [55]. For example, in traditional pastoral regions, the carrying pressure of natural grasslands could be substantially eased in the cool season by feeding with artificial grasses, silage, and crop straw, which was characterized by the decline in livestock-carrying pressure of natural grasslands from 3.8 to 3.1 during 2000-2015 on average [8]. We found consistent results that FSDI_grass_ decreased from 2.8 to 2.54 in the traditional pastoral regions during 2000-2019 (Fig. 2, Fig. 3). We also found a less obvious effect of crop straw in the Tibetan Plateau with an average decrease in FSDI ranging from 0.013 to 0.026. The reason may be that the provincial data that we used cannot reflect the detailed spatial heterogeneity. Another study that focused on the Tibetan Plateau at the county level found that crop straw fodder could significantly alleviate the high rate of over-grazing in counties with a small area of natural grasslands [26].

In the central and eastern provinces, where rainfed mixed farming systems (MR) dominate the livestock production, bringing crop straw into the forage supply system may help reduce air pollution and greenhouse gas emission caused by straw burning [38]. A review pointed out that the crop straw production in these provinces accounted for more than 80% of the entire country [56]. But so far, only less than 30% of them have been used as forage, which may be one of the factors limiting the balance of forage supply and demand in these regions. One possible explanation for the low utilization of crop straw for forage is their high fiber content, low mineral and protein content, poor palatability [26,57], and the infeasibility of removing crop straw within a very short window between harvesting and planting a subsequent crop, particularly in multi-cropping systems in southeastern China [38]. Therefore, the technical improvement in crop straw harvest and conversion plays an important role in improving its nutritional value, digestibility, and overall utilization.

With rising demand for livestock products and forage, China may face even greater challenges in balancing forage supply and demand considering the multiple trade-offs of grazing, protecting grassland ecosystems, and urbanization. Researchers have found that the current meat production per area of grassland in China is only 30% of the world's average [4], and the increasing demand for livestock products (∼16%−30% across all scenarios), would domestically require ∼3–12 Mha of additional pasture between 2020 and 2050 [6], which means there will be additional pressure on natural grasslands to satisfy forage demand. However, higher grazing pressure could result in higher biomass extractions from pastures and arguably lead to widespread land degradation [5]. As our results focused on 2000 to 2019, the PCCs and TCC_gap_ in traditional pastoral and agropastoral regions continued to increase, while those in southern provinces had a downward trend (Figs. S1 and S5). The regional differences suggested that it is likely necessary to adopt targeted forage supply strategies in different regions.

### 4.2 Possible solutions for balancing forage supply and demand

**Fig. 6:**
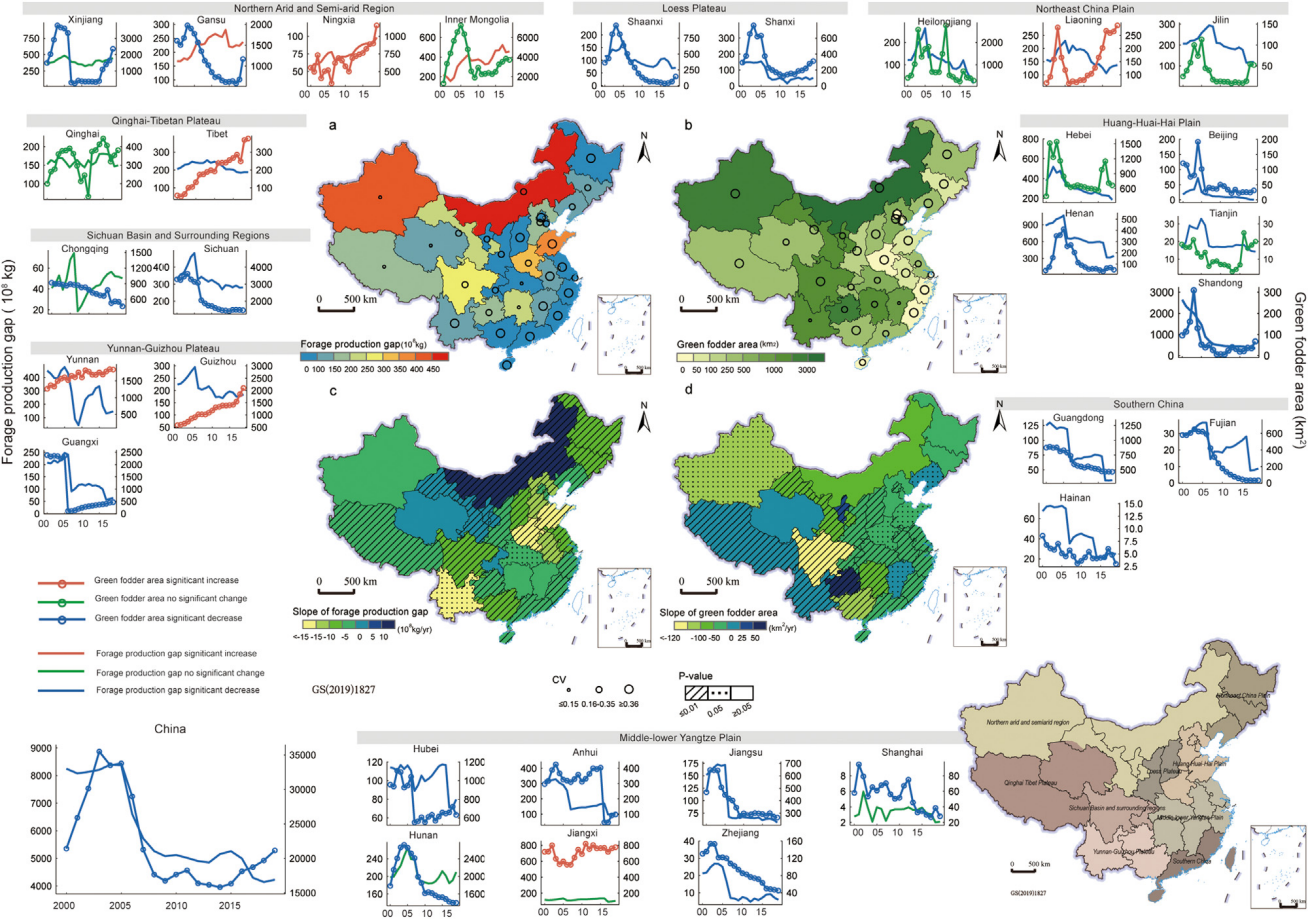
**Provincial and national forage production gaps (a, c) and green fodder sown area (b, d) in 2019 and their changes during 2000–2019.** The outer line charts showed forage production gap (10^8^ kg) and green fodder area (km^2^) in each province and in the whole country during 2000–2019. There are nine patterns of temporal trends of forage production gaps and green fodder area, namely (1) a significant decrease in forage gaps and increase in green fodder area (Liaoning, Guizhou, and Yunnan Provinces, and Tibet); (2) no significant change in forage gaps but a significant increase in green fodder area (Jiangxi Province); (3) both a significant increase in forage gaps and green fodder area (Ningxia Province); (4) both a significant decrease in forage gaps and green fodder area (Beijing, Shanxi, Jiangsu, Zhejiang, Anhui, and Fujian Provinces); (5) no significant change in forage gaps but a significant decrease in green fodder area (Shanghai, Hunan, Chongqing, and Xinjiang Provinces); (6) significant increase in forage gaps but decrease in green fodder area (Gansu Province); (7) significant decrease in forage gaps but no change in green fodder area (Tianjin, Hebei, Heilongjiang, and Jilin Provinces); (8) both no significant change in forage gaps and green fodder area (Qinghai Province); and (9) a significant increase in forage gaps but no significant change in green fodder area (Inner Mongolia).

The introduction of external energy into local forage supply is a promising way to satisfy the growing demand for forage. Researchers have suggested to diversify the feed base and combine multi-sourced forage, including improved natural pastures, straw crops, and forage crops [12,55]. As one of the external sources, green fodder is an important resource that can help fill the forage gap, alleviate natural grassland degradation, and increase livestock production [11,48]. We further investigated the relationships between forage production gaps (kg) and green fodder area from official statistical data (Fig. 6, Text S3). Without considering cross-provincial forage product delivery, we found that the patterns in pastoral provinces had large regional variations, and Gansu Province was the most noteworthy.

In Gansu Province, the supply from natural grasses and crop straws was insufficient to meet the demand for forage (Fig. 2, Fig. 3), and the sown area of green fodder has decreased annually (Fig. 6). This downward trend is not conducive to balancing forage supply and demand, and the forage supply structure needs to be adjusted. Also, it needs to be noted that the forage demand in Inner Mongolia rapidly increased (1, 454 million kg/yr of forage production gap and 2.21 million SU/yr of TCC_gap_), while the green fodder area fluctuated substantially over the 20 years and had an indistinctive downward trend. The scenarios in Guizhou, Yunnan, and Tibet were relatively satisfied by the increased green fodder area and had a decrease in forage gaps, and forage supply and demand may be balanced in the future with further expansion of green fodder area.

In 2016, the Ministry of Agriculture formally proposed a national plan for the structural adjustment of the planting industry, which aimed to promote a “grain-cash-forage” three-dimensional planting structure, ensure ecological security in grasslands, and develop grass-based livestock husbandry [7]. Many studies have demonstrated the economic and ecological benefits of artificial grasslands. In terms of the economic benefits, studies have pointed out that in the same climatic conditions, the production of cultivated artificial forage was 2.7–12.1 times that of natural grasslands [48,58]. If 10% of the areas with suitable water resources and environmental conditions were transitioned to artificial grasslands, then at most 100% of the forage demand could be met [7,59,60].

In terms of the ecological benefits, large areas of natural grassland can be liberated to alleviate the grazing pressure and restore the ecological function through the construction of artificial grasslands [61], which is important for agropastoral and ecologically fragile regions [62], [63], [64]. Researchers have also noted the importance of scientific planning and the spatial-temporal allocation of natural grasslands and artificial grasslands as well as replacing the severely degraded natural grasslands with efficient artificial grasslands [61].

Furthermore, interregional redistribution of forage products is important for enriching the local forage reserve and reducing a dependency on international trade. In 2016, China imported 1.72 million tons of green fodder from overseas to meet the domestic demand. Such a dependence on the international market may introduce substantial risks due to unexpected increases in tariffs and global supply shortages [65]. Recent research predicted that the reliance on imports is projected to increase in China to meet the rapidly increasing demand for livestock products, and that the import of livestock products accounted for 86% of the predicted agricultural trade-embedded green-house gas emissions [6]. Therefore, it is important to enhance China's self-sufficiency in forage and livestock production by encouraging forage industry development and facilitating intra-national redistribution. Enhancing the availability, accessibility, and affordability of China's livestock forage is conducive not only to addressing the conflict of food demand between humans and animals [12,55], but also to improve the overall ecosystem services [66] and alleviate the dependence of China's feed and livestock products on international market.

### 4.3 Uncertainties and limitations

We investigated forage supply and demand in China at the provincial scale during 2000–2019, and we proposed expanding crop straw and green fodder as possible solutions to satisfy the increasing demand for forage. However, our findings are confined by some uncertainties and limitations in the absence of more refined spatiotemporal data.

First, the inter-annual changes in the feeding ratios of straw and cross-regional forage trades may cause uncertainties. Studies have shown that crop straw and artificial fodder resources in China are unevenly distributed spatially [67], and that inter-provincial transactions may exist. Because of the unavailability of long-term official data on the feeding ratios of straw (f) and interregional forage trade, we did not take these factors into account in our estimations [68]. At the national scales, imported fodder products from abroad increased rapidly from 1.87 billion kg to 7.99 billion kg during 2000–2019 (Fig. S6), which also helped alleviate the contradiction between forage supply and demand. But our assessments were based on local forage supply-demand relationships at the national and provincial scales. This uncertainty may lead to an exaggeration of the relationships between PCC and TCC in some provinces.

Second, we did not exclude the TCC_grass_ in natural protected areas from the total TCC_grass_ because of its very small percentage (∼5%, Fig. S4). But for Qinghai Province, the TCC_grass_ in protected areas accounted for 30% of the total TCC_grass_. Although the natural grasslands in protected areas mainly feed wildlife, entirely excluding livestock might be difficult in some areas where indigenous people raise animals for sustenance [69]. The migration of wild herbivores could lead to competition among grazing wildlife and livestock for food, water, and space in grassland ecosystems [17]. For example, Yang et al. (2017) found wild large herbivore increased the carrying pressure of natural grasslands in Maduo County, Qinghai Province [70]. We also did not consider the conversion from immature livestock to standard sheep units, because detailed data on young livestock was not available. The overestimation of the PCC of domestic livestock partially offset the underestimation of the PCC of wild herbivores.

Third, our assessments on forage supply and demand were confined to the provincial scale because long-term and pixel-scale datasets on natural grasslands and green fodder were not available. On the one hand, the natural grassland dataset that we used was produced in the 1980s, and the lack of consideration of land use change associated with grasslands may also lead to underestimation of TCC_grass_ after large-scale implementation of ecological programs over the decades. However, annual high quality grassland maps are hard to obtain because there are large uncertainties in the grassland area estimates from existing land cover products (2.80 to 3.93 million km^2^) [48].

Also, due to the different accuracies among annual land cover maps, interannual variation analyses were not recommended for MODIS land cover products [71]. Therefore, the static grassland resource maps could avoid potential bias caused by the uncertainty in the land cover maps for any year and has been applied in many studies [[72], [73], [74], [75]]. In the future, we will consider developing an algorithm based on existing grassland maps and remote sensing to extract the spatial- temporal pattern of natural grasslands to address this deficiency.

On the other hand, although we have mapped artificial green fodder in Qinghai Province [76], long-term pixel-based green fodder maps covering the entire country were not available. Thus, our current discussions on the contribution of green fodder cannot be measured at the uniformed scale of livestock population (SU). So, we only compared the trends between forage production gaps and green fodder areas.

Forth, a more accurate estimation of PCC needs to consider intra-annual livestock dynamics, especially for non-traditional pastoral regions. Unfortunately, current statistics only count livestock at the middle and end of the year, and datasets on the slaughter cycles and rates are hard to obtain. A recent study in Gannan County of Gansu Province estimated the monthly PCC using the stocking number of livestock at the end of the year, the slaughter and survival rate of young livestock from statistical data, and field surveys of local livestock breeding and slaughter dynamics [77]. At present, it is difficult to conduct a nationwide large-scale questionnaire on intra-annual livestock dynamics. In the future, we will consider to improve the PCC assessment for non-traditional pastoral regions by considering the slaughter dynamics.

Therefore, more detailed studies that use datasets from multiple sources and nationwide, large-scale field surveys are needed to better understand the inter-province and international regulations of forage supply-demand relationships.

## 5 Conclusion

We determined the current state and spatial patterns of the practical and theoretical carrying capacities of forage over the past 20 years and identified the gaps in theoretical carrying capacity and forage production under two scenarios, with and without considering utilization of crop straw. We found significant spatial heterogeneity among the different provinces in China. At the national scale, the TCC_gap_ and FSDI_crop+grass_ decreased by 48.7% and 47.5% during 2000–2019, respectively, due to the decreased PCC and increased TCC_grass_ and TCC_crop_. At the provincial scale, large forage gaps were found in traditional pastoral and agropastoral provinces, especially in Inner Mongolia, with a gap of 69.66 million SU in theoretical carrying capacity in 2019 and a rate of increase of 2.21 million SU/yr. Agricultural provinces had great potential for the feed conversion of crop straw, with the increased TCC_crop_ and imbalance between crop straw production and forage feeding ratios. Our study highlighted the urgency of adopting regional targeted strategies for expanding forage sources, including improving the technical conversion of crop straw into fodder and the cultivation of green fodder crops to balance the forage supply and demand in China.

## Declaration of competing interest

The authors declare that they have no conflicts of interest in this work.

## Appendix Supplementary materials

Image, application 1
