# Supplementary material for: A large forage gap in forage availability in traditional pastoral regions in China
Source: Fundam Res. 2023 Jan 12;3(2):188–200. doi: 10.1016/j.fmre.2023.01.003 (PMC11630677; doi:10.1016/j.fmre.2023.01.003)
Supplement: Supplementary file 1 [file mmc1.docx]

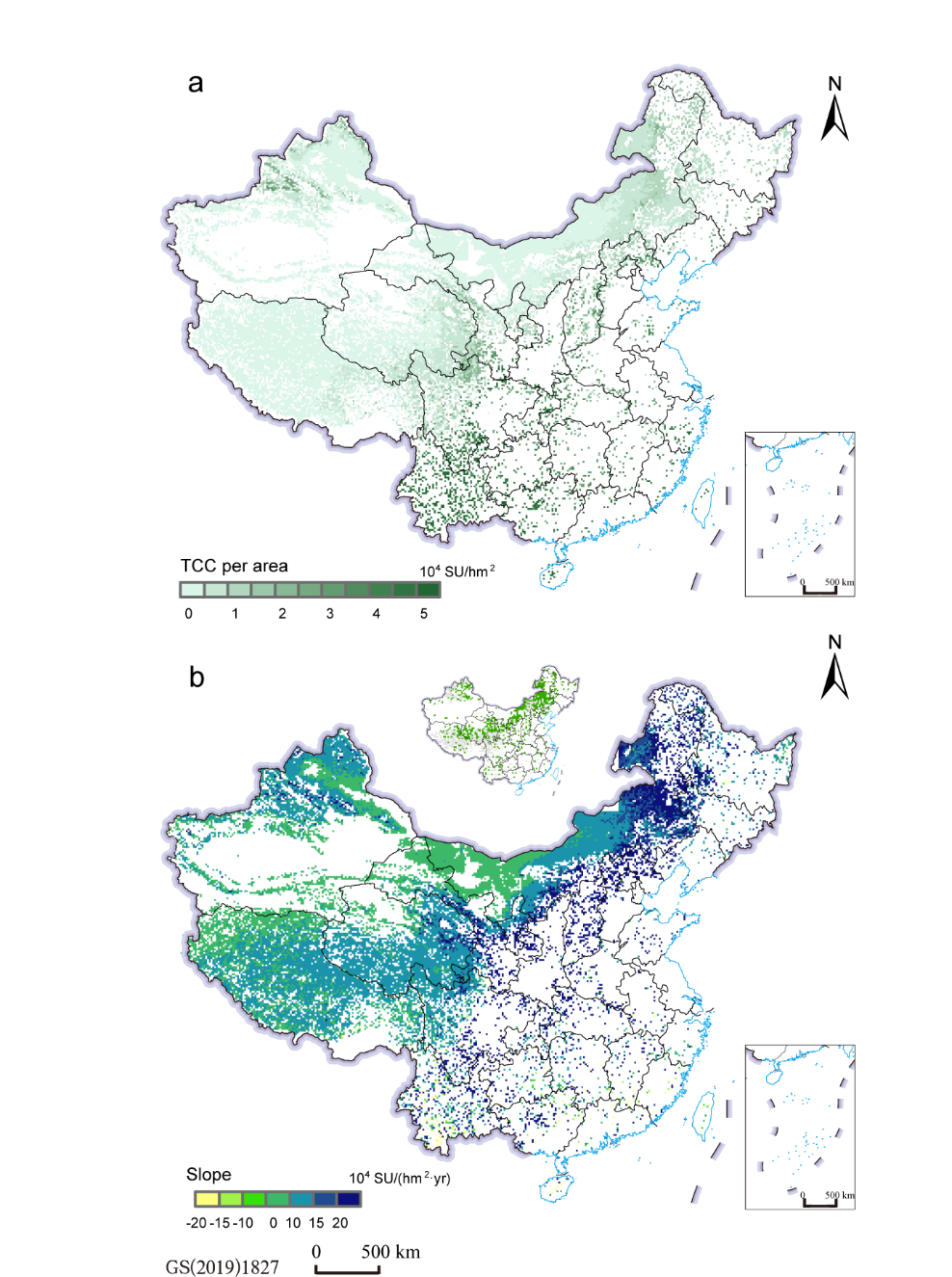


**Fig. S1** Theoretical carrying capacity per area in 2019 **(a)** and pixel-based dynamic trend during 2000-2019 **(b).** The green pixels in the small plot in Fig. b indicate significance through 0.05.


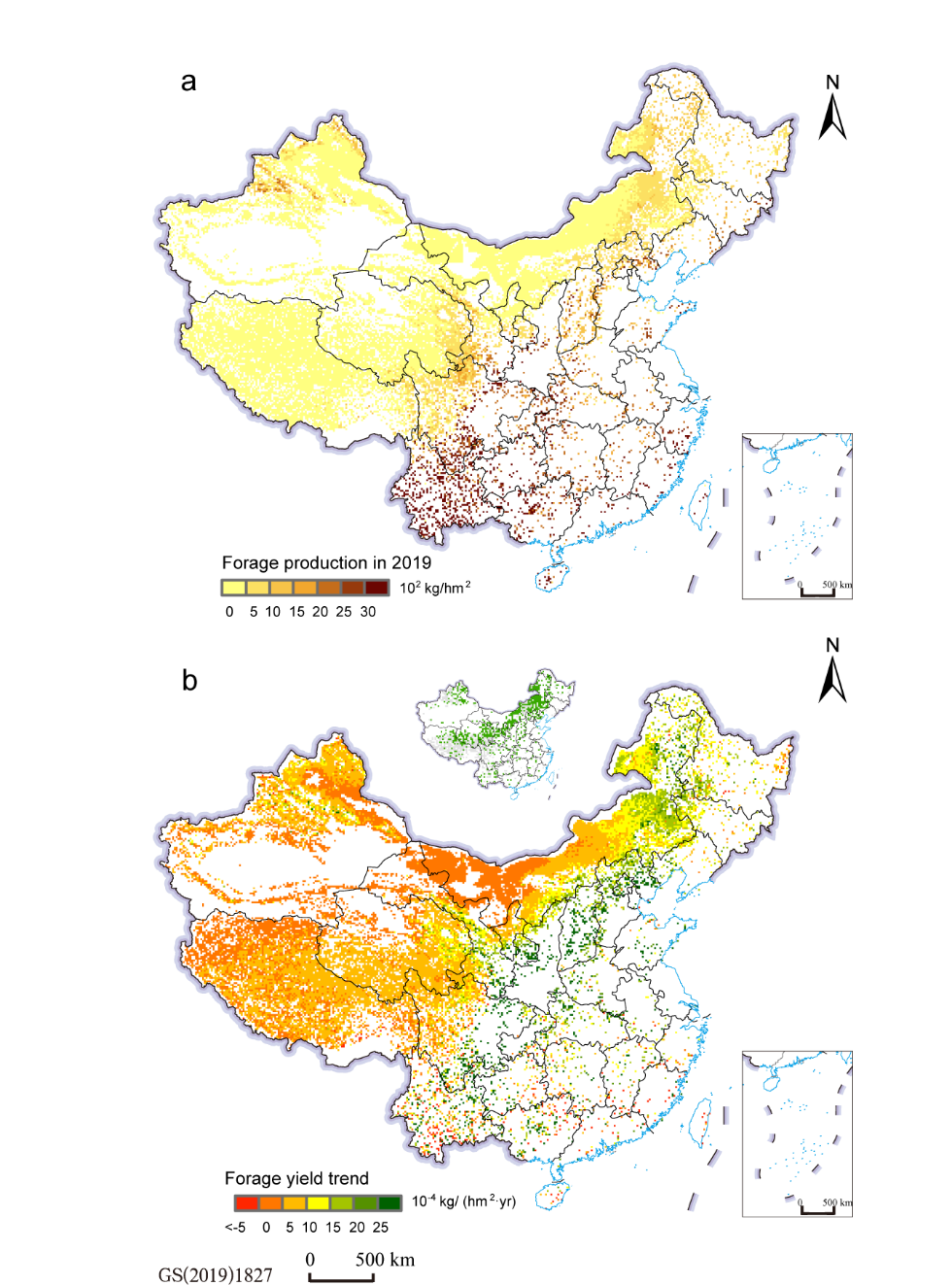


**Fig. S2** Available forage production in 2019 **(a)** and pixel-based dynamic trend during 2000-2019 **(b).** The green pixels in the small plot in Fig. b indicate significance through 0.05.


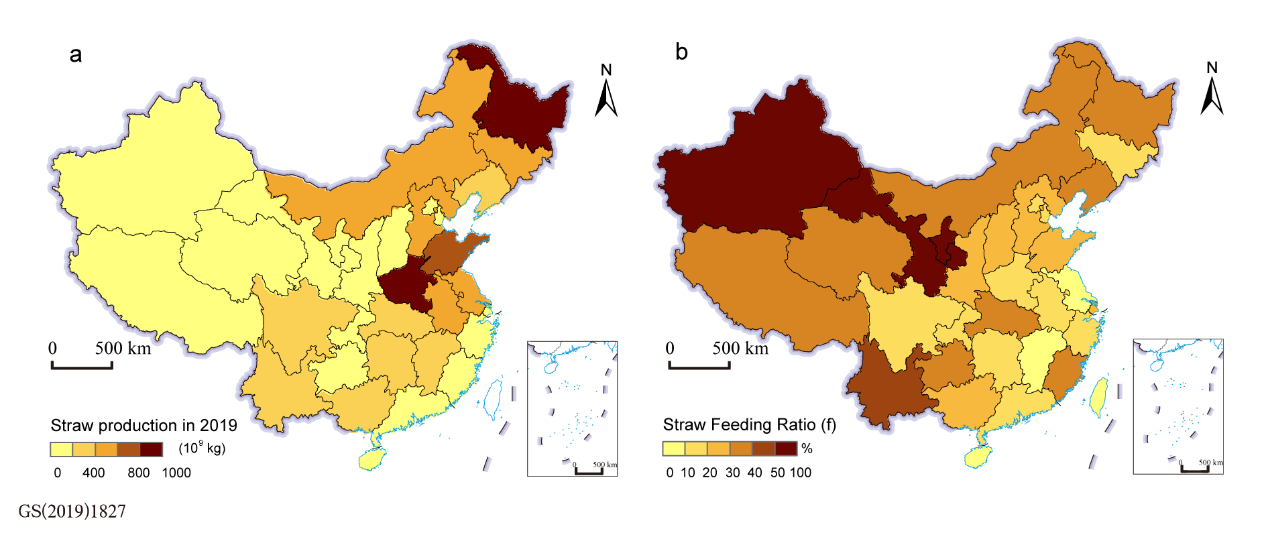


**Fig. S3** Straw production in 2019 **(a)** and feeding ratios in each province **(b).**


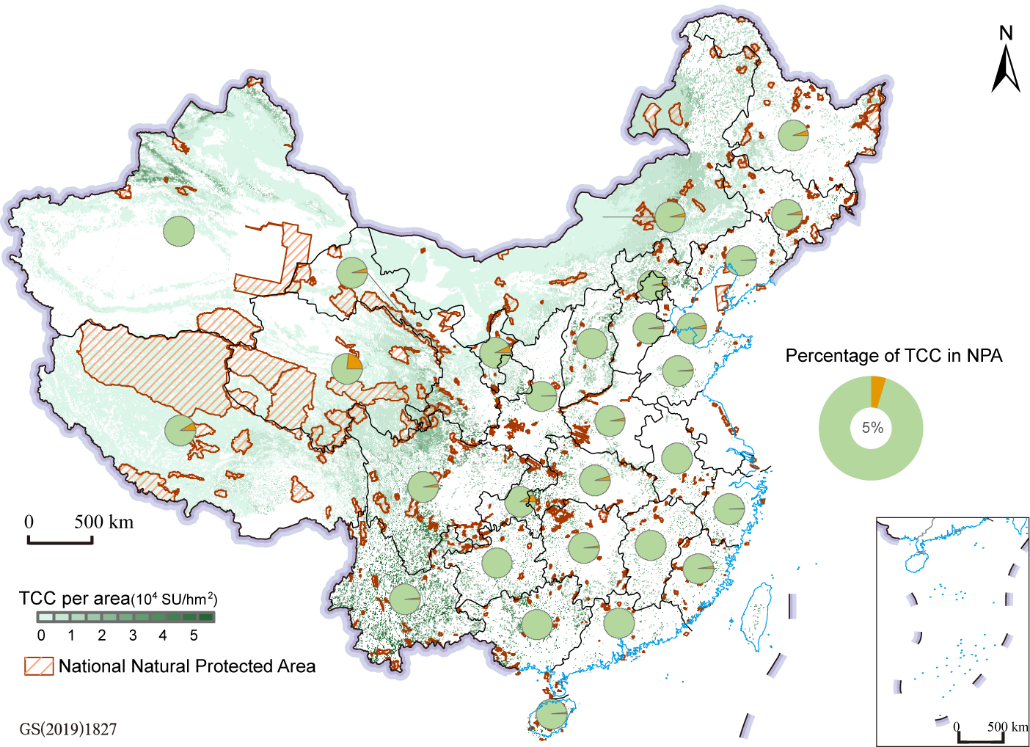


**Fig. S4** The locations of natural protected areas (NPA) and the percentages of TCC_grass_ in natural protected areas.


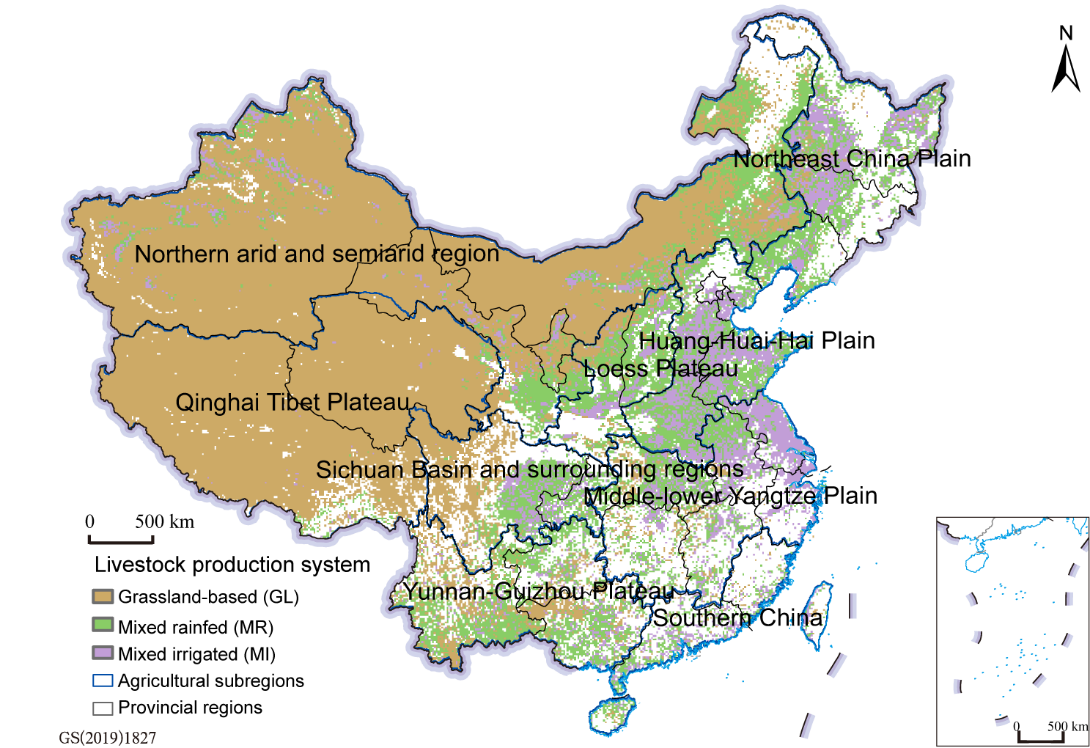


**Fig. S5** The regional division of agricultural subregions, provincial regions, and livestock production systems of China. The agricultural subregions of China from Resource and Environment Science and Data Center (<https://www.resdc.cn/data.aspx?DATAID=275>). The livestock production system map was from Global distribution of ruminant livestock production systems V5 of FAO [1].

**Fig. S6** The quantity of imported fodder and feed products of China from 2000-2019. The data was from the Food and Agriculture Data of FAO (<https://www.fao.org/faostat/zh/#data/TCL>).

**Table S1. Parameter query table for Root/shoot ratio (r), regeneration rate (a), utilization rate (UR_ng_), and standard hay conversion coefficient (K)**

| Class  Code | Class Name | r | a | UR_ng_ | K | Reference |
| --- | --- | --- | --- | --- | --- | --- |
| 0 | Non-grass type | 0 | 0 | 0 | 0 |  |
| 1 | Warm meadow-steppe type | 7.0 | 0.15 | 0.5 | 1 | [2] |
| 2 | Warm steppe type | 6.4 | 0.15 | 0.45 | 1 | [2] |
| 3 | Temperate desert-steppe type | 6.8 | 0.05 | 0.4 | 0.9 | [2] |
| 4 | Alpine meadow- steppe type | 5.8 | 0 | 0.45 | 1 | [3] |
| 5 | Alpine steppe type | 6.2 | 0 | 0.4 | 0.95 | [2] |
| 6 | Alpine desert-steppe type | 5.47 | 0 | 0.35 | 0.9 | [4] |
| 7 | Temperate steppe-desert type | 7.62 | 0.05 | 0.3 | 0.9 | [5] |
| 8 | Temperate desert type | 3.04 | 0.05 | 0.3 | 0.9 | [5] |
| 9 | Alpine desert type | 6.4 | 0 | 0 | 0.9 | [6] |
| 10 | Warm-temperate tussock type | 3.83 | 0.2 | 0.5 | 0.85 | [4] |
| 11 | Warm-temperate shrub tussock type | 1.69 | 0.2 | 0.5 | 0.85 | [5] |
| 12 | Tropical tussock type | 3.58 | 0.2 | 0.55 | 0.8 | [4] |
| 13 | Tropical shrub tussock type | 3.58 | 0.2 | 0.55 | 0.8 | [4] |
| 14 | Arid-tropical shrub tussock scattered with trees type | 3.71 | 0.2 | 0.55 | 0.8 | [5] |
| 15 | Lowland saline meadow type | 4.9 | 0 | 0.5 | 0.95 | [4] |
| 16 | Temperate montane meadow type | 3.5 | 0.15 | 0.55 | 1 | [2] |
| 17 | Alpine meadow type | 6.5 | 0 | 0.5 | 1.05 | [2] |
| 18 | Marsh type | 3.54 | 0 | 0.25 | 0.85 | [4] |
| 19 | Improved grassland | 4.93 | 0.15 | 0.5 | 1.2 | [7] |

**Table S2. The straw-to-grain ratios (S_G_) of crops**

| Crop | Straw-to-grain ratio (S_G_) | Straw |
| --- | --- | --- |
| Rice | 0.9 | Rice straws |
| Wheat | 1.1 | Wheat Straws |
| Corn | 1.2 | Corn stalks |
| Sorghum | 1.6 | Sorghum stems |
| Millet | 1.6 | Millet straws |
| Soybean | 1.6 | Soybean stalks |
| Potato | 0.5 | Potato plants |
| Peanut | 0.8 | Peanut vines |
| Rapeseed | 1.5 | Rapeseed stems |
| Sunflower | 3 | Sunflower stalks |
| Sugarbeet | 0.1 | Sugarbeet stems and leaves |
| Vegetable | 0.1 | Vegetable vines |
| Sugarcane | 0.06 | Sugarcane leaves |

**Table S3. Straw feeding ratios (*f*) in each province****^a^**

| Province | *f* | Reference | Province | *f* | Reference |
| --- | --- | --- | --- | --- | --- |
| Beijing | 0.13 | [8] | Hubei | 0.30 | [9] |
| Tianjin | 0.10 | [10] | Hunan | 0.15 | [11] |
| Hebei | 0.25 | [12] | Guangdong | 0.13 | [13] |
| Shanxi | 0.20 | [14] | Guangxi | 0.27 | [15] |
| Inner Mongolia | 0.34 | [16] | Hainan | 0.09 | [17] |
| Liaoning | 0.35 | [18] | Chongqing | 0.19 | [19] |
| Jilin | 0.19 | [20] | Sichuan | 0.17 | [21] |
| Heilongjiang | 0.35 | [22] | Guizhou | 0.35 | [23] |
| Shanghai | 0.26 | [13] | Yunnan | 0.46 | [13] |
| Jiangsu | 0.05 | [24] | Tibet | 0.34 | [25] |
| Zhejiang | 0.13 | [26] | Shaanxi | 0.24 | [27] |
| Anhui | 0.11 | [28] | Gansu | 0.54 | [29] |
| Fujian | 0.33 | [30] | Qinghai | 0.30 | [31] |
| Jiangxi | 0.01 | [32] | Ningxia | 0.54 | [33] |
| Shandong | 0.22 | [34] | Xinjiang | 0.53 | [31] |
| Henan | 0.12 | [35] |  |  |  |

^a^ The data were retrieved from the start of art studies on the feeding ratios of crop straw in each province.

**Table S4. Grazing time in warm seasons**

| Province Code | Province Name | Average warm days^a^ | Province Code | Province Name | Average warm days |
| --- | --- | --- | --- | --- | --- |
| 110000 | Beijing | 221.55 | 420000 | Hubei | 319.43 |
| 120000 | Tianjin | 241.80 | 430000 | Hunan | 322.34 |
| 130000 | Hebei | 231.46 | 440000 | Guangdong | 356.02 |
| 140000 | Shanxi | 232.82 | 450000 | Guangxi | 349.94 |
| 150000 | Inner Mongolia | 200.70 | 460000 | Hainan | 360.05 |
| 210000 | Liaoning | 212.30 | 500000 | Chongqing | 325.85 |
| 220000 | Jilin | 199.67 | 510000 | Sichuan | 308.56 |
| 230000 | Heilongjiang | 191.45 | 520000 | Guizhou | 318.96 |
| 310000 | Shanghai | 318.70 | 530000 | Yunnan | 340.55 |
| 320000 | Jiangsu | 306.19 | 540000 | Tibet | 191.05 |
| 330000 | Zhejiang | 322.28 | 610000 | Shaanxi | 275.88 |
| 340000 | Anhui | 311.88 | 620000 | Gansu | 220.39 |
| 350000 | Fujian | 334.61 | 630000 | Qinghai | 175.96 |
| 360000 | Jiangxi | 323.35 | 640000 | Ningxia | 235.35 |
| 370000 | Shandong | 264.96 | 650000 | Xinjiang | 224.44 |
| 410000 | Henan | 297.37 | 710000 | Taiwan | 360.05 |

^a^ Average warm days were represented as the average number of days with stable temperatures above 5°C in each province from 2000 to 2020, and 365 minus the warm season grazing time was used as the cold season grazing time.

**Table S5. Abbreviation glossary**

| Abbreviation | Full name |
| --- | --- |
| PCC | Practical carrying capacity |
| TCC_grass_ | Theoretical carrying capacity of natural grasslands |
| TCC_crop_ | Theoretical carrying capacity of crop straws |
| TCC_gap_ | Theoretical carrying capacity gaps that considering forage supplied from both natural grasslands and crop straws |
| FSDI_grass_ | Forage supply and demand index only supplied from natural grasslands |
| FSDI_crop+grass_ | Forage supply and demand index considering supplied from both natural grasslands and crop straws |

**Text S1. Methodology for determining the number of grazing days in warm season of each province**

Climatic conditions are one of the important environmental factors for livestock survival and vegetation growth [36]. The number of grazing days in the warm season is usually determined by regional climate conditions and forage resources and regulated by local livestock characteristics, customs, and other factors [37, 38]. To determine the number of grazing days, we used the indicator of agricultural threshold temperature. Agricultural threshold temperature marks the temperature at which a certain weather phenomenon begins, ends, or changes, and is often divided by 0℃, 5℃, 10℃ and 20℃ [39, 40]. The number of days with temperature ≥5℃ is the length of growing season for overwintering crops, early spring crops, and most natural vegetation [41]. When the daily average temperature in spring is stable through 5°C, the natural grassland enters the green grass season and produce organic matter for livestock feeding. And when the daily average temperature in autumn is lower than 5°C, the natural grassland enters senescence season, and is no longer suitable for grazing [36-38]. Considered that the most suitable survival temperature of herbivorous livestock is above 5-8 °C for most of the major large livestock [36], we finally determined the number of grazing days in warm season as the number of temperature stable through 5°C.

We used the five-day sliding average method to calculate the number of temperature stable through 5°C. It is a commonly used statistical method to estimate the initial date and end date of the stable through the boundary temperature, and it is also the most suitable method for China 's climate characteristics [42]. Specifically, the date of the first of the five-day sliding average temperatures that steadily passed 5°C was determined as the initial day, the last as the final day, and the duration days between the initial and final day was the length of the warm season [41]. The number of warm season days were averaged for each province from 2000-2019.

The daily average air temperature dataset was obtained from more than 2,400 meteorological stations observed at meteorological element stations in China from 2000 to 2019 (<https://www.resdc.cn/data.aspx?DATAID=230>). The in-situ temperature data was averaged in 371 municipal administrative regions across the country and then aggregated to the provincial scale.

**Text S2. The provincial heterogeneity of crop straw production and feeding ratios**

We found mismatch between crop straw production and straw feeding ratios in China. To be specific, provincial regions where crop straw production was abundant tended to have lower feeding ratios. For example, the production of crop straw in Henan and Shandong Provinces was over 8 billion kg, but only less than 30% of them were fed for livestock. Conversely, the production of crop straw in Xinjiang, Ningxia, Qinghai, and Tibet were less than 3 billion kg, but over 40%-50% of them were used as forage for supplementary feeding (Fig. A3). These regional differences led to a complex spatial distribution of the carrying capacity of crop straw.

**Text S3. Supplementary information on the spatial and temporal dynamics of green fodder area**

As supplementary information, we analyzed the green fodder area and its dynamics during 2000-2019 in all provinces (Fig. 6). On a national level, the sown area of green fodder crops accounted for less than 2% of the total area of farm crops in China in the past two decades, and this percentage has declined to below 1% since 2012 [43, 44]. The demand for forage showed a downward trend during 2000-2019, while the green fodder area increased before 2004 and rapidly decreased during 2005-2015, and then slowly increased after 2016. On a provincial level, we found green fodder area was huge in northern pastoral provinces and southwestern provinces. In 2019, Inner Mongolia had the largest sown area of green fodder at 3712 km^2^, followed by Xinjiang Province (2356 km^2^) and Guizhou Province (2103 km^2^). In most of the eastern provinces, the green fodder area was smaller than 100km^2^ (Fig. 6b). During 2000-2019, we found a general decline of the green fodder area in almost all provinces except Qinghai, Tibet, Ningxia, Yunnan, Guizhou, Jiangxi, and Jilin provinces (Fig. 6d). Among the provinces with increasing trends, the green fodder area in Guizhou Province has increased the fastest at the speed of 65.91 km^2^/a, followed by Ningxia Province at the speed of 25.06 km^2^/a in the past 20 years. At the same time, the green fodder area in Sichuan Province decreased 50% from 3285.9 to 1488.7 km^2^ rapidly. These implied the great potential of grasslands in mountain areas of southern China [45].

**References**

[1] T. P. Robinson, P. K. Thornton, G. Franceschini, et al., Global livestock production systems. In (2011).

[2] A. Ma, G. Yu, N. He, et al. Above-and below-ground biomass relationships in China’s grassland vegetation. Quaternary Sciences 34(4) (2014) 769-776.

[3] Y. Yang, J. Fang, C. Ji, et al. Above‐and belowground biomass allocation in Tibetan grasslands. Journal of Vegetation Science 20(1) (2009) 177-184.

[4] H. H. Shen, Y. K. Zhu, X. Zhao, et al. Analysis of current grassland resources in China. Chinese Science Bulletin 61(2) (2016) 139-154.

[5] J. Fan, H. Zhong, W. Harris, et al. Carbon storage in the grasslands of China based on field measurements of above-and below-ground biomass. Climatic Change 86(3) (2008) 375-396.

[6] Y. Yang, J. Fang, W. Ma, et al. Large-scale pattern of biomass partitioning across China\"s grasslands. global ecology & biogeography 19(2) (2010) 268-277.

[7] G. Tian, B. Xu, X. Yang, et al. Using MODIS time series data to estimate aboveground biomass and its spatio-temporal variation in Inner Mongolia's grassland between 2001 and 2011. International Journal of Remote Sensing 34(21-22) (2013) 7796-7810.

[8] F. Teng, L. Yang, B. Xiong, et al. Investigation and Research on Comprehensive Utilization of Crop Straw in Beijing City. Agricultural Engineering (2016).

[9] M. Ye, Z. Ding, Z. Deng, et al. Analysis on the status and comprehensive utilization of straw resources in Hubei Province. Journal of Anhui Agricultural Sciences 49(17) (2021) 78-81.

[10] W. Li and S. Zhang. Fiscal Policy Study on Promoting Comprehensive Use of Straw in Tianjin. Contemporary Rural Finance and Economics 12 (2018) 41-47.

[11] J. Zhang and X. Shi. Present situation and suggestion of crop straw utilization in Hunan Province. Modern Agricultural Science and Technology 06 (2019) 135-138.

[12] H. Yi, This year, the comprehensive utilization rate of straw in Hebei will reach 96%, and the structure is still unreasonable. In Observer (2017).

[13] G. Zhang, F. Lu, H. Zhao, et al. Residue usage and farmers' recognition and attitude toward residue retention in China's croplands. Journal of Agro-Environment Science 36(5) (2017) 981-988.

[14] H. Yang, Study on the utilization status of crop straw resources in Shanxi. In College of Biotechnology, Shanxi University: (2016) Vol. Master's degree.

[15] T. Pang, X. Yi, Y. Liang, et al. Research report on the current situation and development and utilization of forage resources in Guangxi. Feed Research 10 (2017).

[16] Y. Yao, F. S. Zhang, C. H. Wang, et al. Crop Straw Utilization in Typical Agricultural and Pastoral Areas. Journal of Inner Mongolia University for Nationalities(Natural Sciences) (2016).

[17] Hainan "13th Five-Year" straw comprehensive utilization implementation plan. In D. o. A. o. H. Province, Ed. (2017).

[18] X. Gao. Present situation, problems and suggestions of straw comprehensive utilization in liaoning province. Horticulture & Seed 40(11) (2020) 60-62.

[19] G. L. Liu Ke, Tang Ning, Wei Xiuli, Li Ping, Li Hong, Zhang Kai, Yang Yupeng. Present situation and prospect of comprehensive utilization of crop strawin Chongqing. China Biogas 36(03) (2018) 87-91.

[20] Y. Zhu, W. Na, D. Xi, et al. Current situation survey of Comprehensive Utilization of Crop Straw in Jilin Province. Modern Agricultural Science and Technology 14 (2020) 160-161.

[21] B. Xian, P. Wang and Y. Tang. Current situation and problems of comprehensive utilization of straw resources in Sichuan Province. Sichuan Agricultural Science and Technology 04 (2018).

[22] C. Li. Crop straw comprehensive utilization status and solution strategy. World Tropical Agriculture Information 09 (2021) 9-10.

[23] L. Gao, L. Ma, W. Zhang, et al. Estimation of nutrient resource quantity of crop straw and its utilization situation in China. Transactions of the Chinese Society of Agricultural Engineering 25(7) (2009) 173-179.

[24] K. Zhang, Technical evaluation on the comprehensive utilization of straw in Jiangsu. In Nanjing Agricultural University: (2016) Vol. Master.

[25] G. Li, W. Sun, H. Zhang, et al. Balance between actual number of livestock and livestock carrying capacity of grassland after added forage of straw based on remote sensing in Tibetan Plateau. Transactions of the Chinese Society of Agricultural Engineering 30(17) (2014) 200-211.

[26] Y. Qiu, B. Ye and R. Lu. Analysis and discussion on the current situation of crop straw fodder utilization in Zhejiang Province. Journal of Zhejiang Agricultural Sciences 59(02) (2018) 331-332.

[27] J. Zhu, R. Li, Z. Zhang, et al. Temporal and spatial distribution of crops straw and its comprehensive utilization mechanism in Shaanxi. Nongye Gongcheng Xuebao/Transactions of the Chinese Society of Agricultural Engineering 29(25) (2013) 1-9.

[28] Q. Fan, Research on circulation agriculture model based on comprehensive utilization of straw in Anhui Province. In School of Resources and Environment, Anhui Agricultural University: (2020) Vol. Master.

[29] F. Yuan. Analysis of the current situation and countermeasures of comprehensive utilization of crop straw in Gansu. Agriculture Engineering Technology(Agricultural Product Processing Industry) 12 (2012).

[30] B. S. Tang, Y. U. De-Yi and Y. Q. Huang. Straw Resources and Their Utilization in Fujian. Acta Agriculturae Universitis Jiangxiensis (2003).

[31] J. Bao, J. Yu, Z. Feng, et al. Situation of distribution and utilization of crop straw resources in seven western provinces, China. Yingyong Shengtai Xuebao 25(1) (2014).

[32] L. Jiang, Z. Huang, J. Xiong, et al. Current situation and consideration of comprehensive utilization of crop straw in Jiangxi Procince. Jiangxi Science 38(06) (2020) 891-895.

[33] H. Huang and T. Sun, Research report on the comprehensive utilization of crop straw in Ningxia. In The Communists, X. Bin, Ed. (2018) Vol. 12.

[34] W. Jiang, H. Zhang, N. Li, et al. Status of crop straw utilization and equipments in Shandong Province. Journal of Chinese Agricultural Mechanization (2019).

[35] W. Zheng, Research on resource utilization of crop straw in Henan Province. In Henan Normal University: (2018) Vol. Master.

[36] S. L. Li, L. F. Wang, Q. L. Song, et al. Analysis of Animal Husbandry Climate Resources in Daxing ' anling Region. Heilongjiang Meteorology 04 (2002) 28-35.

[37] B. Ge. An Analysis of Animal Climatic Resources of Gansu Province. Gansu meterology 13(1) (1995) 18-22.

[38] G. Z. Peng. Characteristics of changes in the date of boundary temperature and climatic resources for animal husbandry on the northwest Sichuan Plateau. Resources and Environment in the Yangtze Basin 24(4) (2015) 617-624.

[39] F. Xiuzao and T. Bingyan, Principle of agricultural meteorology, China Meteorological Press, Beijing, 1994.

[40] X. Zhang, X. Pan, X. U. Lin, et al. Spatio-temporal variation of agricultural thermal resources at different critical temperatures in China's temperate zone. Resources Science (2017).

[41] Z. Wang, S. Zang, D. Zhou, et al. Temporal and Spatial Variation of Agricultural Threshold Temperature from 1957 to 2016. Scientia Geographica Sinica 40(1) (2020) 137-148.

[42] S. T. Wang. Statistical Method for the Beginning and Ending Dates of Daily Mean Temperature Stably Passing through the Threshold Temperature. Meteorological Monthly 06 (1982).

[43] S. Zhai, Q. Chen and W. Wang. What drives green fodder supply in China?-A Nerlovian analysis with LASSO variable selection. Sustainability (Switzerland) 11(23) (2019).

[44] Q. H. Chen, G. S. Liu and Y. M. Liu. Can product-information disclosure increase Chinese consumer's willingness to pay for GM foods? The case of Fad-3 GM lamb. China Agricultural Economic Review 9(3) (2017) 415-437.

[45] S. Gao, R. Duan, H. Wang, et al. Farming-Pastoral Ecotone of Northern China Plays Important Role in Ensuring National Food Security. Bulletin of the Chinese Academy of Sciences (6) (2021) 643-651.
